# Monolobar Caroli's Disease and Cholangiocarcinoma

**DOI:** 10.1155/1999/70985

**Published:** 1999-07

**Authors:** Eddie K. Abdalla, Christopher E. Forsmark, Gregory Y. Lauwers, J. Nicolas Vauthey

**Affiliations:** 1 Department of Surgery University of Florida Gainesville Florida USA; 2 Department of Medicine Division of Gastroenterology University of Florida Gainesville Florida USA; 3 Department of Pathology University of Florida Gainesville Florida USA

## Abstract

Caroli's Disease (CD) is a rare congenital disorder characterized by cystic dilatation of the intrahepatic bile ducts. This report describes a patient with cholangiocarcinoma arising in the setting of monolobar CD. In spite of detailed investigations including biliary enteric bypass and endoscopic retrograde cholangiography, the diagnosis of mucinous cholangiocarcinoma (CCA) was not made for almost one year. The presentation, diagnosis and treatment of monolobar CD and the association between monolobar CD and biliary tract cancer are discussed. Hepatic resection is the treatment of choice for monolobar CD.


*HPB Surgery*, 1999, Vol. 11, pp. 271–277
Reprints available directly from the publisher
Photocopying permitted by license only

© 1999 OPA (Overseas Publishers Association) N.V.
Published by license under
the Harwood Academic Publishers imprint,
part of The Gordon and Breach Publishing Group.
Printed in Malaysia.

### *Case Report*

### Monolobar Caroli's Disease and Cholangiocarcinoma

EDDIE K. ABDALLA [a], CHRISTOPHER E. FORSMARK [b], GREGORY Y. LAUWERS [c]
and J. NICOLAS VAUTHEY [a,*]

[a] *Department of Surgery;*
[b] *Department of Medicine, Division of Gastroenterology;*
[c] *Department of Pathology, University of Florida, Gainesville, Florida*

*(Received 7 February 1997)*

**Caroli's Disease (CD) is a rare congenital disorder characterized by cystic dilatation of the intrahepatic bile ducts. This report describes a patient with cholangiocarcinoma arising in the setting of monolobar CD. In spite of detailed investigations including biliary enteric bypass and endoscopic retrograde cholangiography, the diagnosis of mucinous cholangiocarcinoma (CCA) was not made for almost one year. The presentation, diagnosis and treatment of monolobar CD and the association between monolobar CD and biliary tract cancer are discussed. Hepatic resection is the treatment of choice for monolobar CD.**

*Keywords:* Caroli's disease, cholangiocarcinoma, biliary cystic disease

### INTRODUCTION

Caroli's Disease (CD) is a rare congenital condition characterized by diffuse or focal cystic dilatation of the intrahepatic bile ducts [1]. Two forms are described, a simple type in which bile ducts are dilated and cystic, without hepatic fibrosis or its sequelae. The second type is associated with peri-portal fibrosis and leads to portal hypertension, esophageal varices and sometimes liver failure. Both types are characterized by recurrent episodes of intrahepatic biliary lithiasis and cholangitis as well as increased risk for development of biliary tract cancer. In some patients, the disease is confined within one hepatic lobe, with less than 150 cases of monolobar CD reported in the literature [2, 3].

The etiology of CD is unclear, but it has been postulated that prenatal vascular accident leads to stricture and dilatation of bile ducts [1]. Liver resection, biliary bypass, endoscopic or percutaneous drainage, alone or in combination, have all been used to treat CD [4–6].

This report describes a patient with CD confined to the left liver, who developed recurrent cholangitis after biliary enteric bypass and required left lobectomy. At the time of lobectomy, the patient was found to have metastatic, low-grade nucinous cholangiocarcinoma. The presentation, diagnosis and treatment of mono-

*Corresponding author. Tel.: (713) 792-2022, Fax: (713) 792-0722, e-mail: jvauthey@notes.mdacc.tmc.edu lobar CD and the association between monolobar CD and biliary tract cancer is discussed.

### CASE REPORT

A 73-year-old man was referred with recurrent cholangitis four weeks following laparoscopic cholecystectomy. Intraoperative cholangiogram had demonstrated multiple hepatic duct defects and left intrahepatic duct dilatation. Physical examination was unremarkable at the time of referral, and laboratory data were as follows: total bilirubin 2.1 mg/dl, direct bilirubin 1.1 mg/dl, AST 126 U/l ALT 111 U/l, alkaline phosphatase 648 U/l, WBC 15,000/mm$^3$. Abdominal ultrasound confirmed persistent intrahepatic bile duct dilatation and common bile duct obstruction, but no liver mass.

Surgical exploration revealed a normal appearing liver with a remnant of the gallbladder infundibulum and long cystic duct with an inflammatory node obstructing the common bile duct. There was no hepatic mass palpable or identified by intraoperative ultrasound. Intraoperative choledochoscopy showed mild ductal inflammation and a single left intrahepatic stone, which was extracted. Frozen and permanent sections of the common hepatic duct and liver parenchyma revealed no tumor or cirrhosis. End-to-side hepaticoduodenostomy was performed to provide adequate drainage and retain endoscopic access to the biliary tree. The patient was discharged after five days.

The patient returned within one year with recurrent cholangitis. Biliary scan revealed normal excretion of the right lobe of the liver with delayed excretion of the left liver. Computed tomography scan demonstrated persistent multifocal dilatation confined to the left bile duct system without mass (Fig. 1). Endoscopic retrograde cholangiography showed cystic dilatation of the left bile duct system without evidence of obstruction and a normal right biliary tree. The choledochoduodenostomy was patent. Brushing cytology was negative. Magnetic resonance cholangiography confirmed patent intra-and extrahepatic bile ducts with ductal dilatation confined to the left liver. Given the persistent cholangitis and that disease was confined to a single hepatic lobe, left lobectomy was indicated.

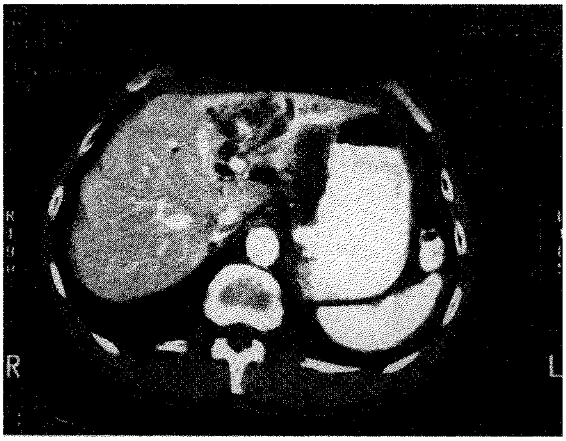

FIGURE 1 CT scan of the abdomen reveals dilated left biliary system, normal right biliary system and no evidence of a mass or tumor.

At operation, sheets of mucinous tumor were found, though restricted to the dome of the liver and right hemidiaphragm. Frozen section revealed low-grade adenocarcinoma. There was no other implant or lymphadenopathy found upon further exploration of the abdomen. A palliative left hepatic lobectomy was performed. Given the low-grade nature of the neoplasm, the peritoneum of the right diaphragm and the hepatic capsular implants were resected to debulk all gross disease. The post-operative course was unremarkable but for a mild pneumonia which had resolved at discharge on the ninth post-operative day.

On examination of the resected specimen, the hepatic capsule was unremarkable while coronal sections revealed gross ectasia of the left bile duct system without definite obstruction by stone or tumor mass. The ducts were filled with mucin and their walls were thickened. The surrounding hepatic parenchyma was grossly unremarkable.

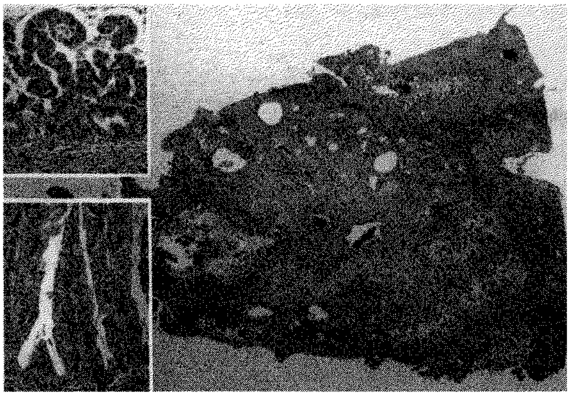

FIGURE 2 Large panel: Low power (1.5X) view of hepatic hilum demonstrating distended bile ducts with prominent papillary lining epithelium. Inserts demonstrate the two observed histologic patterns. Upper insert: Short papillae with marked architectural and cytologic atypia (125X). Lower insert: Taller, well-differentiated papillae with minimal nuclear atypia (125X).

Histopathology revealed diffuse involvement of the bile duct by a well-differentiated papillary mucinous cholangiocarcinoma (Fig. 2). The neoplastic epithelium displayed increased nuclear/cytoplasmic ratio, nuclear hyperchromasia and cigar shaped, tightly packed nuclei. The cytoplasm was filled with mucin and architecturally ranged from flat to papillary. In spite of exhaustive sampling of the specimen, no infiltrative neoplasm was identified. The peritoneal implants were composed of small strips of low-grade epithelium in large pools of mucin.

### DISCUSSION

In 1958, Caroli and Couinaud characterized congenital intrahepatic biliary cystic disease [1]. Later, Barros' review of the literature confirmed two basic types, one which manifests simply with cystic intrahepatic bile ducts, and another associated with hepatic fibrosis [2]. Since then more than 300 cases of CD have been reported, with 137 confined to a single hepatic lobe [3, 6, 7–17].

The pathophysiology of the disease is unknown, though Caroli postulated that it is congenitally acquired [1]. Doppman suggested that prenatal hepatic vascular accident may lead to bile duct stricture and subsequent dilatation [19]. Reports of CD in twin sisters and in families suggest that this disease is inherited [20]. When CD is associated with congenital hepatic fibrosis, it is transmitted as an autosomal recessive disorder [21]. Cholangitis, bile stasis and intrahepatic stone formation are common features of the disease as illustrated in the patient reported. Septicemia, intrahepatic and subphrenic abscess can follow. In the case presented, a single intrahepatic stone was extracted at the time of biliary bypass but cholangitis and bile duct dilatation persisted. The disease usually involves the entire liver but may be restricted to a single lobe or segment. In monolobar CD, the left liver is most commonly affected with a 3:1 left lobe predominance [5]. The reason for this predominant distribution is unknown.

Symptoms occur after 5 to more than 60 years of life. The first episode of bacterial cholangitis is usually spontaneous, and the main (and often only) symptom is unexplained fever without pain or jaundice. If associated with congenital hepatic fibrosis, manifestations of portal hypertension may dominate the clinical presentation. Liver function tests are normal except for moderately elevated alkaline phosphatase and gamma glutamyltranspeptidase [5].

Ultrasound or computed tomography are often the first studies to demonstrate an abnormally dilated biliary system. Cholangiography (percutaneous or endoscopic) defines the biliary anatomy. Hepatobiliary scintigraphy evaluates function. In the patient presented it documented intact biliary excretion of the right lobe.

The most serious complication of CD is the development of biliary tract cancer. Dayton reported cancer in 10 of 142 patients with CD (7%) [6]. Similarly, Guntz, in 1991, found eight cases of malignant degeneration in 104 patients with monolobar CD (7%) [3]. Our review of the literature (Tab. I) revealed 13 cases of carcinoma in 137 cases of monolobar CD (11%). Eight were

TABLE I Reported cases of monolobar CD and biliary tract cancer

Left Monolobar Caroli's Disease and Carcinoma (8 cases)

| Author | Year | Age/sex | Histologic type | Treatment | Outcome |
|---|---|---|---|---|---|
| Todani [42] | 1978 | 26/F | Metastatic cholangiocarcinoma | Excision and hepatico-jejunostomy | Died 48 days after operation |
| Chen [43] | 1981 | 72/M | Cholangiocarcinoma | Left lobectomy | Not reported |
| Leroy [44] | 1982 | 51/F | Papillary mucoid cholangiocarcinoma | Left lobectomy | Not reported |
| Roudot-Thoraval [45] | 1982 | 49/F | Papillary cholangiocarcinoma | Left lobectomy | Not reported |
| Chevillotte [46] | 1984 | 48/M | Papillary cholangiocarcinoma | Left lobectomy and hepaticojeju-nostomy | Died 16 months after operation |
| Guntz [21] | 1987 | 62/F | Gallbladder adenocarcinoma *in situ* | Cholecystect-omy and hepaticojeju-nostomy | Disease free at 58 months |
| Joly [47] | 1990 | 74/M | Cholangiocarcinoma *in situ* | Left lobectomy | Not reported |
| Present case | 1997 | 73/M | Papillary mucinous cholangiocarcinoma | Left lobectomy, peritoneumect-omy | Symptom free at 10 months |

Right Monolobar Caroli's Disease and Carcinoma (5 cases)

| Author | Year | Age/sex | Histologic type | Treatment | Outcome |
|---|---|---|---|---|---|
| Phinney [48] | 1981 | 57/M | Poorly to moderately well-differentiated cholangiocarcinoma | Choledochojej-unostomy × 2 | Died in post-operative period |
| Dayton (case 4) [6] | 1983 | 59/M | Invasive cholangiocarcinoma | Choledochoje-junostomy | Died at 1 month |
| Rossi [28] | 1987 | 64/M | Invasive cholangiocarcinoma | Transhepatic stenting | Died at 4 months |
| Horie [49] | 1987 | 40/M | Papillary cholangiocarcinoma | Right partial lobectomy, cholecystectomy | Symptom free at 3.5 years |
| Falco [17] | 1993 | 68/M | Papillary mucinous cholangiocarcinoma | Hepaticoenter-ostomy | Died at 13 months |

located on the left side and five on the right. All were cholangiocarcinomas except for one case of gallbladder carcinoma [21]. Similar to the case presented, the majority of the patients were men older than 50 suggesting longstanding subclinical disease before the onset of cancer.

The increased risk of cholangiocarcinoma in CD is also found in cystic disease of the extrahepatic bile ducts and oriental hepatolithiasis [22–24]. Cholangiocarcinoma develops in 2.5–28% of bile duct cysts [25–32], whereas the incidence of biliary carcinoma in the absence of biliary cystic disease is only 0.0007–0.014% [25]. The reason for this association remains unknown but bile stasis and chronic cholangitis are considered to be risk factors [26, 33].

The diagnosis of cancer in the setting of CD is difficult. Endoscopic biopsy, with brushing and washing yields a diagnosis in less than half of cases of bile duct cancers. The overall sensitivity of preoperative cytology is 42% or less [34–37]. A mass is infrequently identified. Surgical exploration with frozen and permanent sections is frequently non-diagnostic [6].

The extent of intrahepatic disease and the presence of fibrosis or cirrhosis should guide the treatment of CD. When the disease is localized, resection is the mainstay of treatment [3, 20, 25, 38]. Biliary bypass, whether by choledochoduodenostomy or Roux-en-Y choledochojejunostomy, cannot circumvent the development of hepatolithiasis, recurrent cholangitis, or the risk of cancer [25–29, 38–40]. In the patient presented, the cholangiocarcinoma was a non-invasive, yet aggressive, mucinous tumor which implanted in the peritoneum of the right diaphragm and the capsule of the right lobe. This seeding outside of the biliary tract was likely due to the manipulation, including choledochoscopy, performed at the time of biliary enteric bypass.

Reports persist in the recent literature suggesting that localized disease might be treated palliatively, endoscopically or surgically with biliary enteric bypass, based on the premise that drainage procedures avert the risk of cancer associated with chronic infection and bile stasis [4]. This unfortunately ignores Todani's finding that 57% of 63 cases of carcinoma in patients with all types of biliary cystic disease had previously undergone drainage procedures, and that cancer was found from 1 to 32 years later [38].

Diffuse CD is a more difficult problem. Most recommend a combination of resection and internal bypass, endoscopic or percutaneous stents, or even in rare cases, hepatic transplantation. Antibiotics and litholytic agents are adjuncts [4, 5]. Prognosis for extensive bilateral disease and CD associated with hepatic fibrosis is much worse than for limited disease.

In the patient presented, cholangiocarcinoma remained undiagnosed in spite of bile duct exploration and cytology, and was found 10 months following choledochoduodenostomy. The diagnosis of carcinoma is exceedingly difficult preoperatively and the prognosis is poor if the diagnosis is delayed. In monolobar CD, hepatic resection is the treatment of choice as it provides definitive treatment and may contribute to an early diagnosis of cancer. Non-surgical treatment is the mainstay of management of diffuse disease.

### References

[1] Caroli, J. and Couinaud, C. (1958). Une affection nouvelle, sans doute congénitale, des voies biliaires. La dilatation kystique unilobaire des canaux hépatiques. *Semaines des Hôpitaux Paris*, **34**, 136–142.

[2] Barros, J. L., Polo, J. R., Sanabia, J. *et al.* (1979). Congenital cystic dilatation of the intrahepatic bile ducts (Caroli's Disease): Report of a case and review of the literature. *Surgery*, **85**, 589–592.

[3] Guntz, Ph., Coppo, B., Lorimer, G. *et al.* (1991). La maladie de Caroli unilobaire. Aspects anatomocliniques. Démarche diagnostique et théraputique. A propos de 3 observations personelles et de 101 observations de la litérature. *Journal de Chirurgie (Paris)*, **128**(4), 167–181.

[4] Ciambotti, G. F., Ravi, J., Abrol, R. P. and Arya, V. (1994). Right-sided monolobar Caroli's Disease with intrahepatic stones: nonsurgical management with ERCP. *Gastrointestinal Endoscopy*, **40**(6), 761–764.

[5] Blumgart, L. H. (1994). *Surgery of the Liver and Biliary Tract*, 2nd edn. p. 1193. London: Longman Group Limited.

[6] Dayton, M. T., Longmire, W. P. Jr. and Tompkins, R. K. (1983). Caroli's Disease: a pre-malignant condition? *American Journal of Surgery*, **145**, 41–48.

[7] Orozco, H., Takahashi, T., Garcia-Tsau, G. *et al.* (1991). Partial hepatectomy for Caroli's syndrome. Report of two patients with long-term follow-up. *Hepatogastroenterology*, **38**(1), 33–35.

[8] Knoop, M., Keck, H., Langrehr, J. M. *et al.* (1994). Therapie des unilobularen Caroli-Syndroms durch Leberresektion. *Chirurgie*, **65**(10), 861–866.

[9] Izawa, K., Tanaka, K., Furui, J. *et al.* (1993). Extended right lobectomy for Caroli's disease: report of a case and review of hepatectomized cases in Japan. *Surgery Today*, **23**(7), 649–655.

[10] Orsoni, P., Vandenbossche, D., Boukaya, V. *et al.* (1994). Un cas de maladie de Caroli pure, unilobaire. *Journal de Chirurgie (Paris)*, **131**(12), 532–537.

[11] Morono-Cabajosa, E. A., Celdran-Uriarte, A., Moreno-Caparros, A. *et al.* (1993). Caroli's disease: study of six cases, including one with ephithelial dysplasia. *International Surgery*, **78**(1), 46–49.

[12] Zangger, P., Grossholz, M., Mentha, G. *et al.* (1995). MRI findings in Caroli's disease and intrahepatic pigmented calculi. *Abdominal Imaging*, **20**(4), 361–364.

[13] Mohan, L. N., Thomas, P. G., Kilpadi, A. B. and D'Cunha, S. (1991). Liver atrophy associated with monolobar Caroli's disease. *Hepatobiliary Surgery*, **4**(3), 203–206.

[14] Chlumska, A., Velenska, Z. and Sterbova, B. (1994). Nodularni regenerativni hyperplazie jater. *Casopis Lekaru Ceskych*, **133**(7), 209–212.

[15] Vazquez-Iglesias, J. L., Garcia-Reinoso, C., Arnal, F. *et al.* (1991). Caroli's disease. Presentation of 8 cases studied with ERCP. *Revista Espanola de Enfermedades Digestiva*, **80**(1), 35–40.
[16] Hamy, A., Paineau, J. and Visset, J. (1995). Maladie de Caroli segmentaire gauche découverte en biligraphie per-opératoire...ou comment une cholecystectomie sans problème peut conduire à une hépatectomie gauche. *Journal de Chirurgie* (*Paris*), **132**(3), 114–117.
[17] Falco, E., Nardini, A., Celoria, G. *et al.* (1993). Malattia di Caroli associata a colangiocarcinoma. Un caso de nostra osservazione. *Minerva Chirurgie*, **48**(17), 961–964.
[18] Doppam, J. L., Dunnick, N. R., Girton, M. *et al.* (1979). Bile duct cysts secondary to liver infarcts: report of a case and experimental production by small vessel hepatic artery occlusion. *Radiology*, **130**(1), 1–5.
[19] Ribeiro, A., Reddy, R. K., Bernstein, D. *et al.* (1996). Caroli's syndrome in twin sisters. *American Journal of Gastroenterology*, **91**(5), 1024–1026.
[20] Caroli, J. (1973). Diseases of the intrahepatic biliary tree. *Clinics in Gastroenterology*, **2**, 147–161.
[21] Guntz, M., Lorimer, G., Cronier, P. and Biais, B. (1987). Cancer *in situ* de la vésicule biliaire avec anomalie de la jonction cholédocho-pancreatique associé à une maladie de Caroli unilobaire gauche. *Chirurgie*, **113**(6), 579–592.
[22] Jan, Y. Y., Chen, M. F., Wang, C. S. *et al.* (1996). Surgical treatment of hepatolithiasis: long-term results. *Surgery*, **120**, 509–514.
[23] Chijiiwa, K., Yamashita, H., Yoshida, J. *et al.* (1995). Current management and long-term prognosis of hepatolithiasis. *Archives of Surgery*, **130**, 194–197.
[24] Vauthey, J. N. and Dudrick, P. (1995). Biliary tract cancer. *Current Opinions in Gastroenterology*, **11**, 445–451.
[25] Flanigan, D. P. (1977). Biliary carcinoma associated with biliary cysts. *Cancer*, **40**, 880–883.
[26] Kagawa, Y., Kashihara, S., Kuramoto, S. and Maetani, S. (1978). Carcinoma arising in a congenitally dilated biliary tract. Report of a case and review of the literature. *Gastroenterology*, **74**, 1286–1294.
[27] Bloustein, P. A. (1977). Association of carcinoma with congenital cystic conditions of the liver and bile ducts. *American Journal of Gastroenterology*, **67**, 40–46.
[28] Rossi, R. L., Silverman, M. L., Braasch, J. W. *et al.* (1987). Carcinomas arising in cystic conditions of bile ducts. *Annals of Surgery*, **205**(4), 377–384.
[29] Saito, S., Tsuchida, Y. and Hashizume, T. (1976). Congenital cystic dilatation of the biliary ducts: surgical procedures and long-term results. *Zeitschirft Kinderchirurgie*, **19**, 49–59.
[30] Todani, T., Watanabe, Y., Narasue, M. *et al.* (1977). Congenital bile duct cysts: classification, operative procedures and review of 37 cases including cancer arising from choledochal cyst. *American Journal of Surgery*, **134**, 263–269.
[31] Nagorney, D. M., McIlrath, D. C. and Adson, M. A. (1984). Choledochal cysts in adults: clinical management. *Surgery*, **96**(4), 656–663.
[32] Tsuchiya, R., Harada, N., Ito, T. *et al.* (1977). Malignant tumors in choledochal cysts. *Annals of Surgery*, **186**, 22–28.
[33] Stain, C. S., Guthrie, C. R., Yellin, A. E. and Donovan, A. J. (1995). Choledochal Cyst in the Adult. *Annals of Surgery*, **222**(2), 128–133.
[34] Vauthey, J. N. and Blumgart, L. H. (1994). Recent advances in the management of cholangiocarcinomas. *Seminars in Liver Diseases*, **14**(2), 109–114.
[35] Harell, G. S., Anderson, M. F. and Berry, P. F. (1981). Cytologic bile examination in the diagnosis of biliary duct neoplastic strictures. *American Journal of Roentgenology AJR*, **137**, 1123–1126.
[36] Muro, A., Mueller, P. R., Ferrucci, J. T. and Taft, P. D. (1983). Bile cytology: a routine addition to percutaneous biliary drainage. *Radiology*, **149**, 846–847.
[37] Desa, L. A., Akosa, A. B., Lazzara, S. *et al.* (1991). Cytodiagnosis in the management of extrahepatic biliary stricture. *Gut*, **32**, 1188–1191.
[38] Todani, T., Tabuchi, K., Watanabe, Y. and Kobayshi, T. (1979). Carcinoma arising in the wall of congenital bile duct cysts. *Cancer*, **44**, 1134–1141.
[39] Deziel, D. J., Rossi, R. L., Munson, J. L. and Braasch, J. W. (1985). Cystic disease of the bile ducts. Surgical management and reoperation. *Problems in General Surgery*, **2**(4), 467–480.
[40] Mercadier, M., Chigot, J. P., Clot, J. P. *et al.* (1984). Caroli's Disease. *World Journal of Surgery*, **8**, 22–29.
[41] Trinkl, W., Sassaris, M. and Hunter, F. M. (1985). Endoscopic papillotomy in Caroli's Disease. Twenty year follow-up of a previously reported case. *Endoscopy*, **17**(2), 81–83.
[42] Todani, T., Narusue, M., Watanabe, Y. *et al.* (1978). Management of congenital choledochal cyst with intrahepatic involvement. *Annals of Surgery*, **187**, 272–280.
[43] Chen, K. T. (1981). Adenocarcinoma of the liver. Association with congenital hepatic fibrosis and Caroli's Disease. *Archives of Pathology and Laboratory Medicine*, **105**, 294–295.
[44] Leroy, J. P., Griffe, J., Briere, J. *et al.* (1982). Un nouveau cas de carcinome developée sur maladie de Caroli. *Semaines des Hōpitaux Paris*, **58**(7), 417–419.
[45] Roudot-Thorval, F., Zafrani, E. S., Hannoun, S. *et al.* (1983). Résection hépatique pour maladie de Caroli limitée au foie gauche. Découverte fortuite d'un cholangiocarcinome. *Gastroenterology and Clinical Biology*, **7**(3), 319–320.
[46] Chevillotte, G., Sastre, B., Sahel, J. *et al.* (1984). Maladie de Caroli localisée et associée à un adénocarcinome papillare mucosecrétant. *Presse Medicine*, **13**(18), 1137–1139.
[47] Joly, I., Choux, R., Baroni, J. L. *et al.* (1990). Carcinome *in situ* sur maladie de Caroli localisee. *Gastroenterology and Clinical Biology*, **14**, 90–92.
[48] Phinney, P. R., Austin, G. E. and Kadell, B. M. (1981). Cholangiocarcinoma arising in Caroli's disease. *Archives of Pathology and Laboratory Medicine*, **105**(4), 194–197.
[49] Horie, Y., Hirayama, C. and Katsube, Y. (1987). Case report: bile duct carcinoma in a focal dilatation of the intrahepatic bile duct. *Clinical Radiology*, **38**, 209–211.

### COMMENTARY

The authors are to be commended for their prompt decision to resect the left lobe of the liver when the patient presented again with acute cholangitis. Indeed, in a 70-year-old man with isolated segmental or LOBAR dilatation of intrahepatic duct, cholangiocarcinoma involving the orifice of major branch of intra-hepatic duct should be suspected until proven otherwise. The rule applies to whatever etiology, be it an oriental hepatolithiasis or western Caroli's disease. A good quality CT scan is preferable before common duct exploration, and biopsy of any suspicious area in the ductal wall during choledochoscopy must be encouraged. Serum carcino-embryonic antigen may be elevated in patients with cholangiocarcinoma and if shown to be elevated will give a hint to the possible etiology of isolated segmental dilatation. Performing a bilio-enteric drainage at the common duct level to a ductal system which is dilated only on one side is conceptually wrong because any drainage operation distal to the probable site of obstruction will certainly fail. Constructing a choledochoduodenostomy in the presence of intrahepatic segmental dilatation will induce recurrent acute cholangitis since food reflux into the intrahepatic ductal system will not be able to egress freely back to the duodenum.

Caroli's disease is an uncommon condition. The diagnosis may not be readily made when first seen by a clinician but the principle of surgery for segmental intrahepatic ductal dilatation should be applicable to any etiology.

Dr. S. T. Fan
Department of Surgery
University of Hong Kong
Queen Mary Hospital
HONG KONG
Hong Kong

